# Ciliary IFT80 regulates dental pulp stem cells differentiation by FGF/FGFR1 and Hh/BMP2 signaling

**DOI:** 10.7150/ijbs.27231

**Published:** 2019-08-06

**Authors:** Xue Yuan, Min Liu, Xu Cao, Shuying Yang

**Affiliations:** 1 Department of Oral Biology, School of Dental Medicine, University of Buffalo, State University of New York, Buffalo, NY, United States; 2 Department of Anatomy & Cell Biology, School of Dental Medicine, University of Pennsylvania, PA, United States; 3 Department of Orthopedic Surgery, Johns Hopkins University School of Medicine, Baltimore, MD, United States

**Keywords:** Primary cilia, intraflagellar transport, dental pulp stem cells, fibroblast growth factor signaling, hedgehog signaling, bone morphogenetic protein

## Abstract

Primary cilia and intraflagellar transport (IFT) proteins control a wide variety of processes during development and tissue homeostasis. However, their potential roles in the regulation of stem cell differentiation and tooth development remain elusive. Here, we uncovered the critical roles of ciliary IFT80 in cilia formation and differentiation of dental pulp stem cells (DPSCs). *IFT80*-deficient DPSCs showed reduced fibroblast growth factor receptor 1 (FGFR1) expression, leading to the disruption of FGF2-FGFR1 signaling. We found, during DPSC differentiation, FGF2-FGFR1 signaling induces stress fiber rearrangement to promote cilia elongation, meanwhile stimulates PI3K-AKT signaling to aid Hh/bone morphogenetic protein 2 (BMP2) signaling activation. These signaling pathways and their coupling were disrupted in *IFT80*-deficient DPSCs, causing impaired differentiation. Our findings revealed a novel mechanism that ciliary protein regulates the odontogenic differentiation of DPSCs through FGF/FGFR1 and Hh/BMP2 signaling.

## Introduction

Stem cells, capable of self-renewal and differentiation, are essential for tissue development and homeostasis 1. Tooth development, like many other organs' formation, requires several types of stem cells, which are controlled by different signaling pathways.

Despite our extensive knowledge in tooth formation, very little is known about the stem cells in dental pulp, until Shi, Gronthos, and coworkers isolated dental pulp stem cells (DPSCs) from human dental pulp and defined their properties of clonogenic, self-renewing, rapidly proliferative, and multi-lineage differentiation ability 2, 3. DPSCs give arise to odontoblasts, which are the columnar polarized cells located at the outer edges of dental pulp. Odontoblasts express dentin matrix protein 1 (DMP1) and dentin sialophosphoprotein (DSPP) and produce dentin. Although DPSCs and bone marrow-derived MSCs (BMMSCs) share similarities in the morphology and flow cytometry profiles, DPSCs are more efficient than BMMSCs in producing mineral *in vitro* 4. Recently, DPSCs were identified and isolated from mouse incisors 5, 6, providing an opportunity to determine the signaling pathways that govern DPSC self-renewal and differentiation. To date, the understanding of the maintenance and differentiation of DPSCs are still elusive. Fibroblast growth factor (FGF) signaling was reported to play an essential role in dentinogenesis, and several studies supported that FGF2 promotes the pulp cell proliferation 7-10 and early differentiation 7. However, it was not clear whether FGF2 signaling has a direct function in DPSCs.

Primary cilia are highly conserved microtubule-based organelles that are present on almost all vertebrate cells. Primary cilia were observed in both embryonic and adult stem cells, including DPSCs 11-13, and recent studies suggested that primary cilia regulate stem cell maintenance and lineage determination 12, 14-16. However, none of them examined the roles of IFT proteins in DPSC self-renewal and differentiation.

Formation and function of primary cilia require intraflagellar transport (IFT) proteins along with other ciliary proteins 17, 18. There are 22 identified IFT proteins that form two complexes, IFT-A and IFT-B 19, 20. Mutation of those proteins usually causes cilia defects, and a wide range of diseases called ciliopathies. These disorders target multiple organs, of which bone and tooth are common ones 21-23. IFT80 belongs to IFT-B complex and mutations of IFT80 have been reported in human Jeune asphyxiating thoracic dystrophy (JATD) and short rib polydactyly type III (SRPIII). Previously, we reported that IFT80 promotes osteogenesis 24 and deletion of *IFT80* in osteoblast precursor cells leads to decreased bone mass with impaired osteoblast differentiation through regulating Hh signaling pathway 25. In this study, we further revealed that deletion of ciliary *IFT80* impairs cilia formation and DPSC differentiation via disrupting FGF/FGFR1 signaling and the coupling of FGF2 and Hh/BMP2 signaling.

## Results

### Primary cilia elongate during odontogenesis

**Figure 1:**
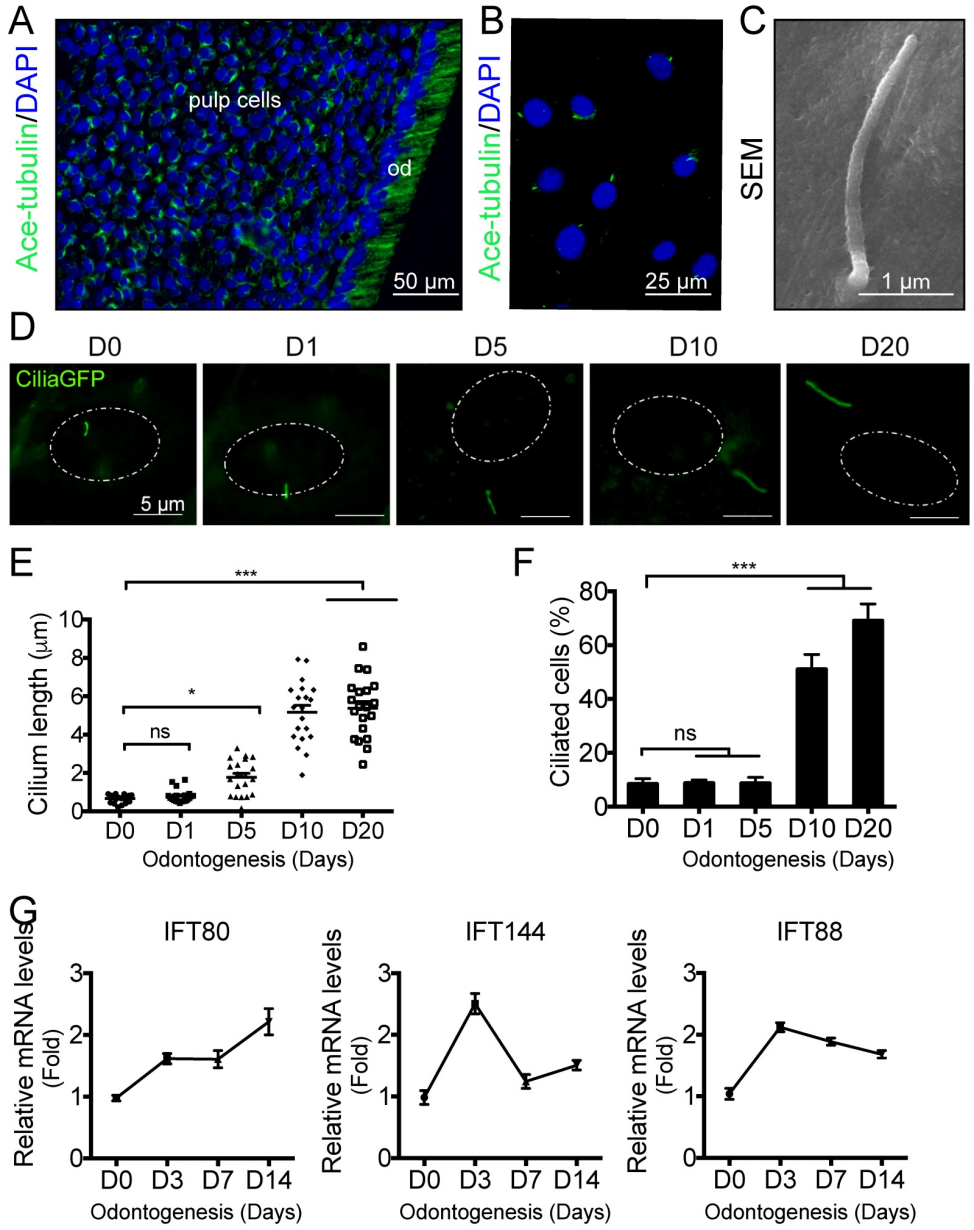
** Primary cilia in DPSCs and cilia elongation during differentiation.** (A) Immunofluorescence analysis of primary cilia in molar sections. Primary cilia were stained with acetylated α-tubulin (green) antibody. DAPI was used for counterstaining. (B) Immunofluorescence analysis of primary cilia on cultured DPSCs. Primary cilia were stained with acetylated α-tubulin (green) antibody. DAPI staining was used as counterstaining. (C) SEM of primary cilia in DPSCs. (D) Immunofluorescence analysis of primary cilia in *CiliaGFP;IFT80^+/+^* DPSCs during odontogenesis. White dot circle represents the nucleus. Scale bars represent 5 μm. (E) Calculated cilia length (n = 20 cells). (F) Calculated cilia percentage (n = 3 with at least 200 cells analyzed). (G) qPCR analysis of *IFT80, IFT144* and* IFT88* expression during DPSC odontogenesis. The expression level of target genes was normalized to *GAPDH* expression (n = 3, triplicates per group). Data are expressed as mean ± SEM; ns, not statistically significant; *p < 0.05; ***p < 0.00001.

Although primary cilia have been visualized on odontoblasts, it is largely unknown whether primary cilia are presented on pulp cells. We stained mouse molar sections with cilia marker acetylated α-tubulin and found that most of the pulp cells displayed primary cilia (Fig. 1A). To examine the functions of primary cilia in dental pulp cells, we isolated primary DPSCs from mouse incisors (Fig. S1A) and characterized the phenotype of DPSCs by cell morphology (Fig. S1B), cell surface markers (Fig. S1C), colony forming ability (Fig. S1D), proliferation rate (Fig. S1E), and multi-lineage differentiation ability (Fig. S1F).

To visualize primary cilia, we isolated DPSCs from *CMV;CiliaGFP* mice, in which, primary cilia were labeled with GFP. Primary cilia were presented on DPSCs (Fig. 1B), and the average length is about 2 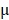
m based on SEM analysis (Fig. 1C). During *in vitro* induced odontogenesis, the length of primary cilia increased (Fig. 1D). Quantification showed that cilia length was significantly longer at day 5 after odontogenic differentiation compared with non-induced ones (Fig. 1E). At day 10, the cilia length reached a peak and any longer inducing time, e.g., 20 days, did not significantly change the cilia length (Fig.1E). We also quantified the percentage of ciliated cells. Before odontogenic inducing, the ciliated cell population was about 10%. This ratio remained the same with 5-day odontogenic induction (Fig. 1F). By day 10, the ciliated cell percentage reached to 50%, and this number increased to 70% by day 20 (Fig.1F). These data suggested that primary cilia might be involved in the odontogenic differentiation of DPSCs.

It is well established that IFT proteins are involved in primary cilia formation. We then asked whether the process of odontogenesis would change IFT protein expression. Using qPCR, we found that the expression of IFT80 was increased along with differentaition (Fig. 1G). However, not all the IFT proteins showed the same pattern. For example, both IFT144 and IFT80 showed the highest expression at day 3 but the expression decreased at a later stage (Fig.1G), suggesting IFT proteins may play different roles in odontogenic differentiation. In this study, we focused on the roles of IFT80 and primary cilia in the odontogenic differentiation of DPSCs.

### Deletion of IFT80 in DPSCs causes cilia loss and odontogenesis defect

**Figure 2:**
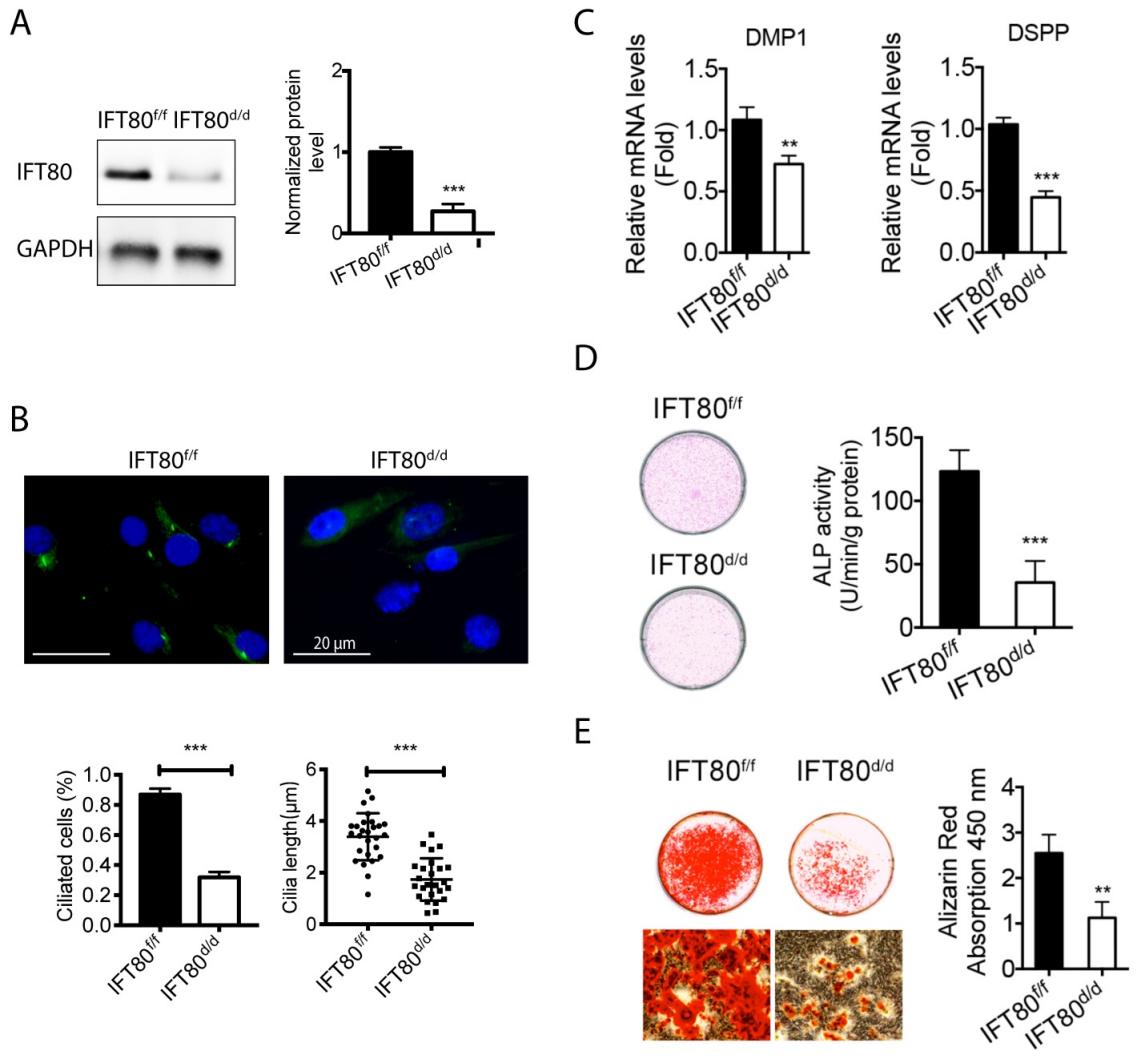
** Deletion of IFT80 disrupts cilia formation and odontogenic differentiation.** (A) Western blot analysis of IFT80 expression in *IFT80^f/f^* and *IFT80^d/d^* DPSCs. IFT80 protein level was normalized to GAPDH (n = 3). (B) Immunofluorescence analysis of primary cilia in cultured DPSCs. Primary cilia were stained with acetylated α-tubulin (green) antibody. DAPI staining was used as counterstaining. Scale bars represent 20 μm. Cilia length (n = 20 cells) and cilia percentage (n = 3 with at least 200 cells analyzed) were calculated. (C) qPCR analysis of *DMP1 and DSPP* expression in *IFT80^f/f^* and *IFT80^d/d^* DPSCs induced with OS medium for 7 days (n = 3, triplicates per group). Target gene expression was normalized to *GAPDH* (n = 3, triplicates per group). (D) ALP staining of *IFT80^f/f^* and *IFT80^d/d^* DPSCs at day 7 of OS induction (n = 3, triplicates per group). (E) Alizarin Red staining of *IFT80^f/f^* and *IFT80^d/d^* DPSCs induced with OS medium for 21 days (n = 3, triplicates per group). Data are expressed as mean ± SEM; ns, not statistically significant; **p < 0.01; ***p < 0.0001.

To further examine the role of IFT80 in DPSCs, we isolated primary DPSCs from *IFT80^f/f^* mice and then infected with adenovirus expressing Cre recombinase to delete *IFT80* (named as *IFT80^d/d^*). Adenovirus expressing the green fluorescent protein (GFP) was used as the control, and the GFP-infected *IFT80^f/f^* DPSCs were still marked as *IFT80^f/f^*. Western blot confirmed Cre adenovirus transduction significantly reduced IFT80 expression in DPSCs (Fig. 2A).

We first noticed that *IFT80^d/d^* DPSCs displayed a defect in cilia formation (Fig. 2B). Quantification results showed that the ciliated population in *IFT80^f/f^* was more than 80%, while it was less than 30% in *IFT80^d/d^* group. A small portion of DPSCs in the *IFT80^d/d^* group showed primary cilia, but the cilia were shorter than that in *IFT80^f/f^* group (Fig. 2B).

We next examined the odontogenic differentiation of DPSCs. To eliminate the effect of proliferation on differentiation, we performed all differentiation assays with a high cell density, at which both *IFT80^f/f^* and* IFT80^d/d^* DPSCs had a minimum proliferation rate (Fig. S2). The result showed that deletion of *IFT80* in DPSCs impaired odontogenic differentiation as evidenced by reduced expression of odontoblast differentiation markers *DMP1* and *DSPP* (Fig. 2C), less ALP activity (Fig. 2D) and decreased mineral nodules formation (Fig. 2E).

### FGF2-FGFR1 signaling is blocked in *IFT80-*deficient DPSCs

**Figure 3:**
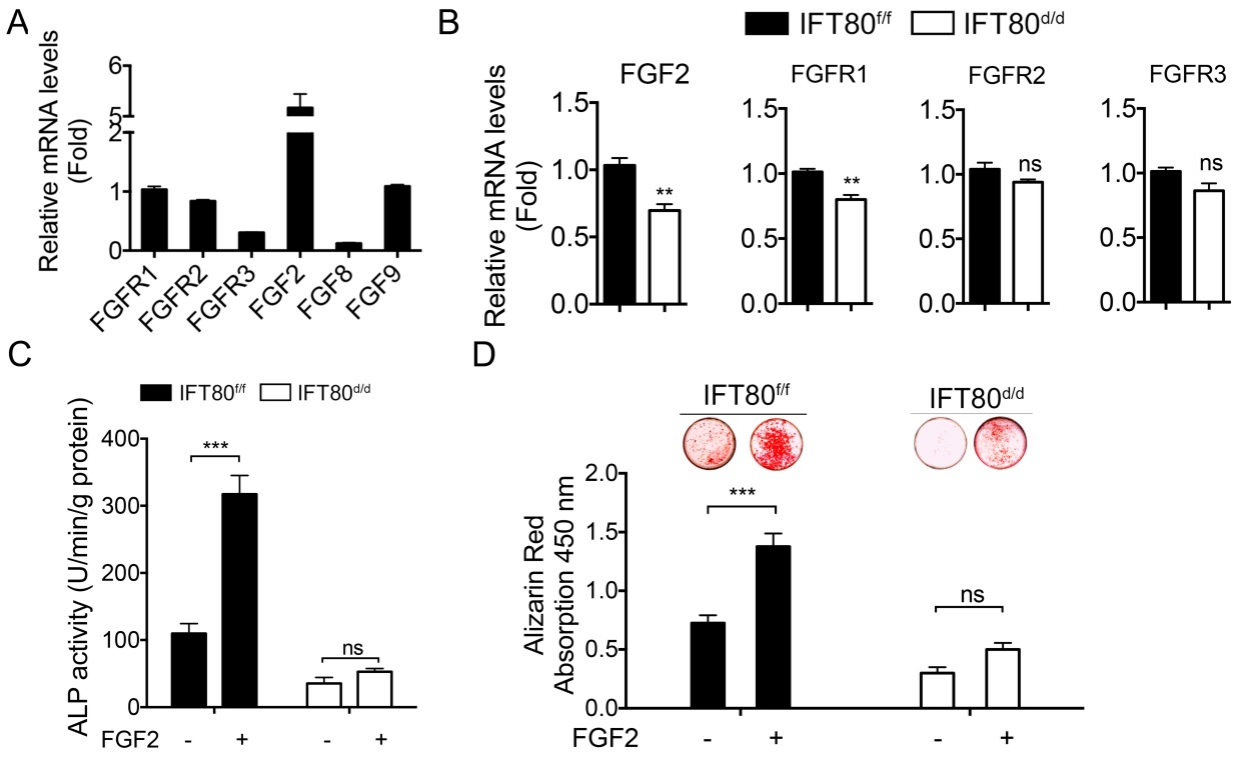
** Deletion of *IFT80* in DPSCs impairs FGF2-FGFR1 signaling.** (A) qPCR analysis of *FGFR1-3*, *FGF2*, *FGF8*, and *FGF9* expression in wild-type DPSCs. The target gene expression was normalized to GAPDH expression (n=3, triplicates per group). (B) qPCR analysis of *FGF2,* and* FGFR1-3* expression in *IFT80^f/f^* and *IFT80^d/d^* DPSCs. The expression levels of *FGF2* and* FGFR1-3* were normalized to *GAPDH* expression (n = 3, triplicates per group). (C) ALP activity of *IFT80^f/f^* and *IFT80^d/d^* DPSCs at day 7 of OS induction with or without FGF2 (10 ng/mL) treatment for the first 3 days (n = 3, triplicates per group). (D) Alizarin Red staining of *IFT80^f/f^* and *IFT80^d/d^* DPSCs at day 14 of OS induction with or without FGF2 (10 ng/mL) treatment for the first 3 days (n = 3, triplicates per group). Data are expressed as mean ± SEM; ns, not statistically significant; ***p < 0.0001.

Our next step was to identify the signaling pathway(s) that IFT80 mediated in DPSCs. FGF signaling regulates the differentiation of many types of cells including dental pulp cells 7, 26. We tested *FGFR1-3*, *FGF2*, *FGF8*, and *FGF9* expression in wild-type DPSCs and found that *FGFR1* and *FGF2* were highly expressed in DPSCs (Fig. 3A). To further examine the mechanism of IFT80 in the regulation of FGF signaling, we studied whether deletion of IFT80 affects the expression of FGFR and FGF. The results showed that the mRNA levels of FGFR1 and FGF2 were dramatically reduced in *IFT80^d/d^* DPSCs compared with these in *IFT80^f/f^* DPSCs (Fig. 3B). Interestingly, *FGFR2* and *FGFR3* expression were not significantly changed (Fig. 3B), suggesting FGF2-FGFR1 is a major FGF signaling affected by *IFT80* deletion.

When* IFT80^f/f^* and* IFT80^d/d^* DPSCs were exposed to FGF2 in the early differentiation stage, we found that FGF2 significantly advanced differentiation in *IFT80^f/f^* DPSCs as tested by ALP activity and Alizarin Red staining (Figs. 3C and 3D). This promotion effect was completely abolished in cells without *IFT80*. These data suggested FGF2 signaling is essential for the differentiation of DPSCs, and IFT80 is involved in FGF2 signaling transduction.

**Figure 4:**
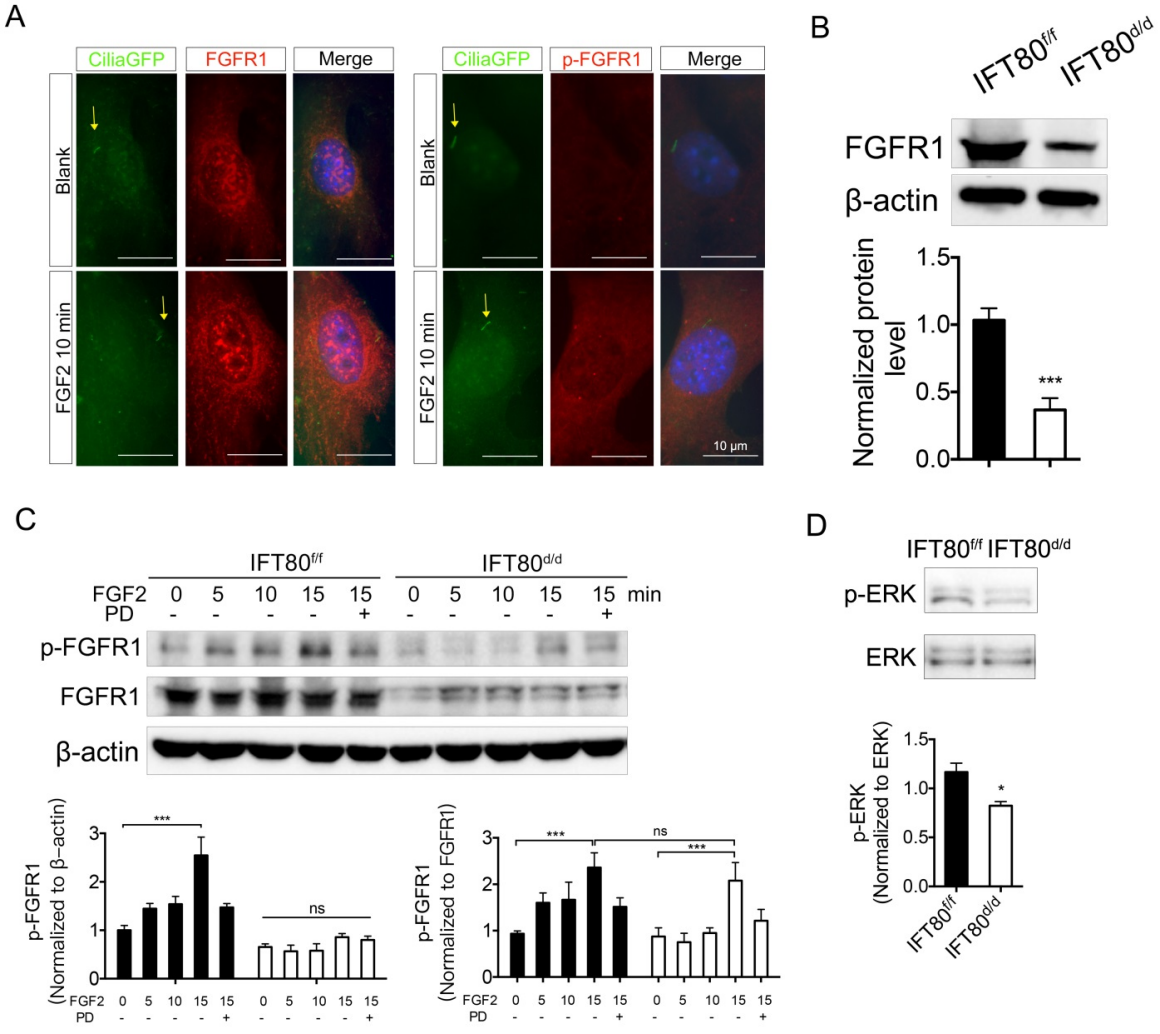
** Deletion of *IFT80* in DPSCs impairs the expression of FGFR1.** (A) *CMV;CiliaGFP* DPSCs were treated with or without FGF2 (10 ng/mL) for 10 min and stained with FGFR1 antibody or p-FGFR1 antibody. The yellow arrows indicate primary cilia (green). DAPI staining was used as counterstaining. Scale bars represent 10 μm. (B) Western blot analysis of FGFR1 expression in *IFT80^f/f^* and *IFT80^d/d^* DPSCs. FGFR1 expression was normalized to β-actin (n = 3). (C) Western blot analysis of FGFR1 phosphorylation with FGF2 (10 ng/mL) stimulation in *IFT80^f/f^* and *IFT80^d/d^* DPSCs. FGFR1 phosphorylation level was normalized to FGFR1 or β-actin (n = 3). PD, PD173074 (1 μM, FGFR1 inhibitor). (D) Western blot analysis of ERK phosphorylation in *IFT80^f/f^* and *IFT80^d/d^* DPSCs. ERK phosphorylation level was normalized to ERK (n = 3). Data are expressed as mean ± SEM; ns, not statistically significant; *p < 0.05; ***p < 0.0001.

To find out how IFT80 was involved in FGF2 signaling transduction, we first asked whether FGFR1, the receptor for FGF2, was located in primary cilia. We examined the location of both FGFR1 and phosphorylated FGFR1 in DPSCs. DPSCs were isolated from *CMV;CiliaGFP* mice, in which primary cilia is labeled with GFP. Immunostaining analysis showed that FGFR1 and the phosphorylation form of FGFR1 (p-FGFR1) in* CMV;CiliaGFP* DPSCs were not located in primary cilia with or without FGF2 stimulation (Fig. 4A), suggesting the reduced FGFR1 expression was not a direct result from cilia loss in *IFT80^d/d^* DPSCs. Immunostaining results also showed reduced FGFR1 expression in *IFT80^d/d^* DPSCs (Fig. 4A). We next used Western blot to confirm the decreased FGFR1 expression in *IFT80*-deficient DPSCs (Fig. 4B).

FGFR1 belongs to the transmembrane receptor tyrosine kinases (RTK) family. FGFs binding induces phosphorylation of tyrosine residues and activates downstream signaling pathways. We found phosphorylation of FGFR1 gradually increased in *IFT80^f/f^* DPSCs upon FGF2 stimulation (Fig. 4C). Pretreated with PD173074, a selective FGFR1 inhibitor, inhibited FGF2-induced FGFR1 phosphorylation in *IFT80^f/f^* DPSCs (Fig. 4C). In contrast, FGF2-induced FGFR1 phosphorylation level was much lower in *IFT80^d/d^* DPSCs when normalized to β-actin. Since *IFT80^d/d^* DPSCs also showed less FGFR1 expression, we also normalized FGFR1 phosphorylation to total FGFR1 expression. We found that the normalized phosphorylation of FGFR1 in *IFT80^d/d^* DPSCs was comparable to that in *IFT80^f/f^* DPSCs 15 min after FGF2 stimulation, suggesting FGFR phosphorylation was not blocked in *IFT80^d/d^* DPSCs. We also found that ERK phosphorylation level reduced, further confirmed the impaired FGFR1 signaling transduction in *IFT80^d/d^* DPSCs (Fig. 4D). Together, these data demonstrated that reduced FGFR1 expression is the primary cause of the FGF2-FGFR1 signaling defect in *IFT80^d/d^* DPSCs.

### FGF2 promotes cilia elongation through actin reorganization in *IFT80^f/f^* DPSCs but not in *IFT80^d/d^* DPSCs

**Figure 5:**
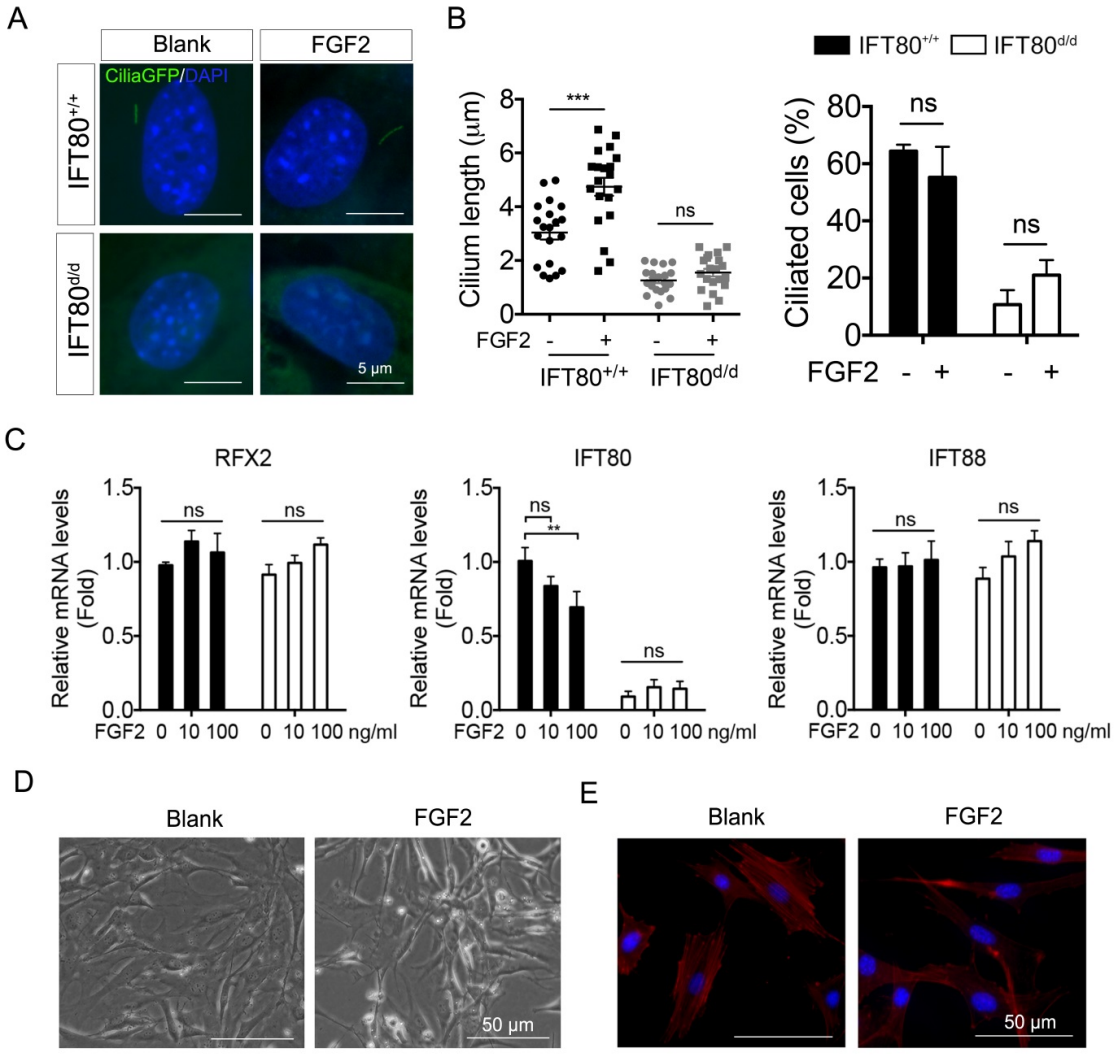
** FGF2 signaling promotes cilia elongation by changing stress fiber.** (A) Analysis of primary cilia in *CiliaGFP;IFT80^+/+^* and *CiliaGFP;IFT80^d/d^* DPSCs treated with or without FGF2 (10 ng/mL). DAPI staining was used as counterstaining. Scale bars represent 5 μm. (B) Calculated cilia length (n = 20 cells) and cilia percentage (n = 3 with at least 200 cells analyzed). (C) qPCR analysis of *RFX2, IFT80* and* IFT88* expression in DPSCs (n = 3, triplicates per group) with FGF2 (10 ng/mL) treatment. (D) Morphology of DPSCs cultured in medium with or without 10 ng/mL FGF2 for 24 hours. (E) DPSCs were stained for actin (red) with or without FGF2 (10 ng/mL) treatment. DAPI staining was used as counterstaining. Scale bars represent 50 μm. Data are expressed as mean ± SEM; ns, not statistically significant; **p < 0.001; ***p < 0.0001.

So far, we have showed that during odontogenesis, primary cilia elongated (Fig. 1) and FGF2 promoted odontogenic differentiation (Fig. 3). We next asked whether FGF2 promotes odontogenic differentiation by increasing cilia length. We analyzed cilia formation in FGF2-treated DPSCs. *Cilia;IFT80^+/+^* and *Cilia;IFT80^f/f^* DPSCs were treated with Ad-CMV-Cre to turn on GFP expression on primary cilia. The results showed that FGF2 significantly induced cilia elongation in *Cilia;IFT80^+/+^* DPSCs without affecting ciliated rate (Fig. 5A). As we expected, FGF2 failed to increase the cilia length and ciliated population in *Cilia;IFT80^d/d^* DPSCs (Fig. 5B), confirming IFT80 was essential for FGF2-induced cilia elongation. We further tested the expression of cilia-related transcription factor *RFX2* and ciliary protein *IFT80* and *IFT88* (Fig. 5C). FGF2 stimulation did not promote the expression of these genes. In contrast, high dose of FGF2 slightly decreased *IFT80* expression (Fig. 5C), suggesting that cilia elongation in FGF2 group was not associated with increased ciliary protein expression. Additionally, we noticed that FGF2-treated DPSCs became small and lost the polygonal cell morphology (Fig. 5D). Furthermore, we found the loss of stress fibers in FGF2 group (Fig. 5E), suggesting the role of FGF2 in actin cytoskeleton reorganization. In our previous study, we revealed that actin reorganization promotes cilia formation 25. Taken these together, FGF promoted cilia elongation through actin reorganization, which consequently promotes differentiation.

### Deletion of IFT80 inhibits DPSC differentiation through inhibiting FGF signaling, Hh signaling and their crosstalk

**Figure 6:**
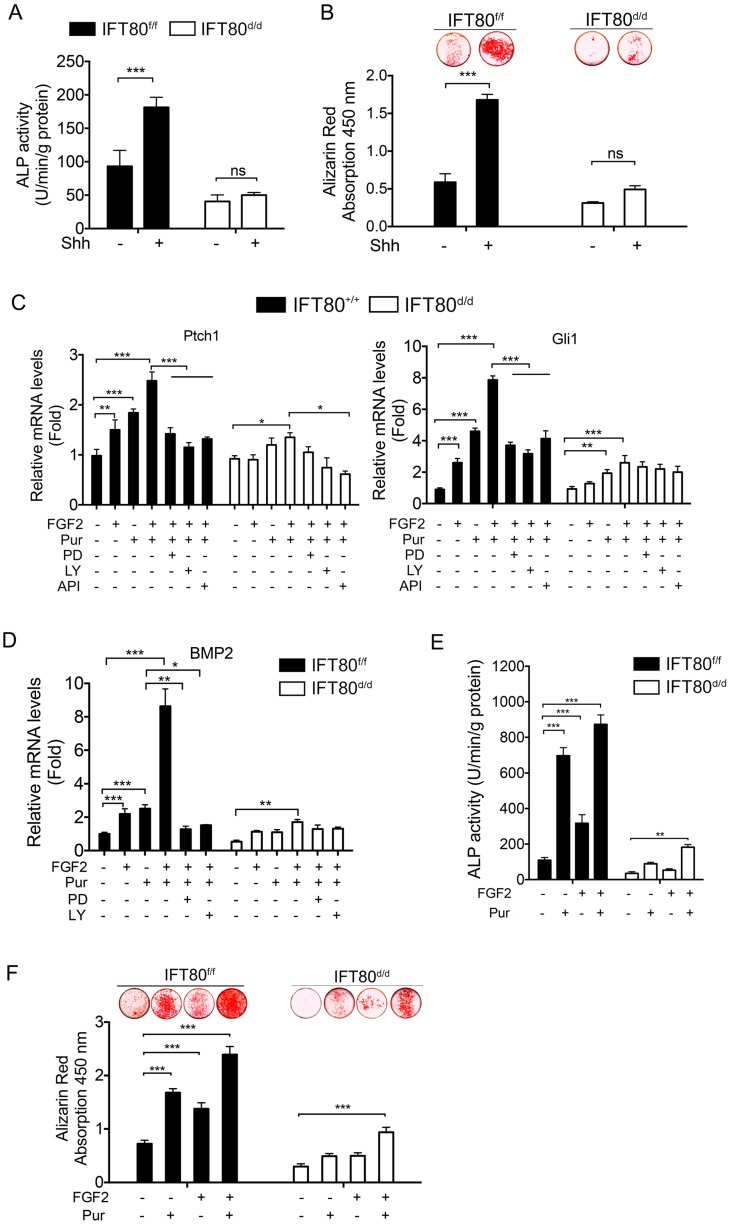
** Crosstalk between FGF2 and Hh/BMP2 signaling during DPSC differentiation is blocked in *IFT80*-deficient DPSCs.** (A) ALP activity of *IFT80^f/f^* and *IFT80^d/d^* DPSCs at day 7 of OS induction with or without Shh (1 μg/mL) treatment (n = 3, triplicates per group). (B) Alizarin Red staining of *IFT80^f/f^* and *IFT80^d/d^* DPSCs at day 14 of OS induction with or without Shh (1 μg/mL) treatment (n = 3, triplicates per group). (C) qPCR results showing *Ptch1* and *Gli1* expression in *IFT80^f/f^* and *IFT80^d/d^* DPSCs. Cells were treated with FGF2 (10 ng/mL), Purmorphomine (Pur, 2 μM), PD173074 (PD, 1 μM), LY294002 (LY, 15 μM) or API-2 (API, 1 µM) as indicated (n=3, triplicates per group). (D) qPCR results showing *BMP2* expression in *IFT80^f/f^* and *IFT80^d/d^* DPSCs. Cells were treated with FGF2 (10 ng/mL), Purmorphomine (Pur, 2 μM), PD173074 (PD, 1 μM), or LY294002 (LY, 15 μM) as indicated (n=3, triplicates per group). (E) ALP activity of *IFT80^f/f^* and *IFT80^d/d^* DPSCs at day 7 of OS induction treated with FGF2 (10 ng/mL) and Purmorphomine (Pur, 2 μM) as indicated (n = 3, triplicates per group). (F) Alizarin Red staining of *IFT80^f/f^* and *IFT80^d/d^* DPSCs at day 21 of OS medium treated with FGF2 (10 ng/mL) and Purmorphomine (Pur, 2 μM) as indicated (n = 3, triplicates per group). Data are expressed as mean ± SEM; ns, not statistically significant; *p<0.05; **p < 0.01; ***p < 0.0001.

It is widely accepted that Hh signaling transduction highly relays on cilia, and Hh signaling is essential for osteogenic differentiation 27, 28. Shh promoted differentiation of *IFT80^f/f^* DPSCs as examined by ALP activity and Alizarin Red staining, but Shh failed to rescue the differentiation defect in *IFT80^d/d^* DPSCs (Fig. 6A and 6B). Since FGF2 promoted cilia formation, we next analyzed whether FGF2 could intensify Hh signaling during differentiation. We found that FGF2 increased *Ptch1* and *Gli1* expression and this effect was enhanced when FGF2 was combined with Purmorphomine, a Hh signaling agonist, in *IFT80^f/f^* DPSCs (Fig. 6C). Inhibition of FGFR1 by PD173074 or inhibition of PI3K-AKT by LY294002 and API-2 significantly blocked FGF2-induced *Gli1* and *Ptch1* expression (Fig. 6C), suggesting FGF2 aided Hh signaling activation through regulating FGFR1 and PI3K-AKT pathway. We further found that FGF2 promoted *BMP2* expression and strongly advanced Purmorphomine-induced *BMP2* expression in *IFT80^f/f^* DPSCs (Fig. 6D). In consistence with these data, FGF2 plus Purmorphomine treatment great promoted differentiation of *IFT80^f/f^* DPSCs (Fig. 6E and 6F). In *IFT80-deficient* DPSCs, the synergistic effect of FGF signaling and Hh signaling was blocked (Fig. 6C-F), highlighting the critical role of IFT80 in the crosstalk of FGF signaling and Hh signaling.

### FGF2 promotes BMP2 signaling through PI3K-AKT signaling

**Figure 7:**
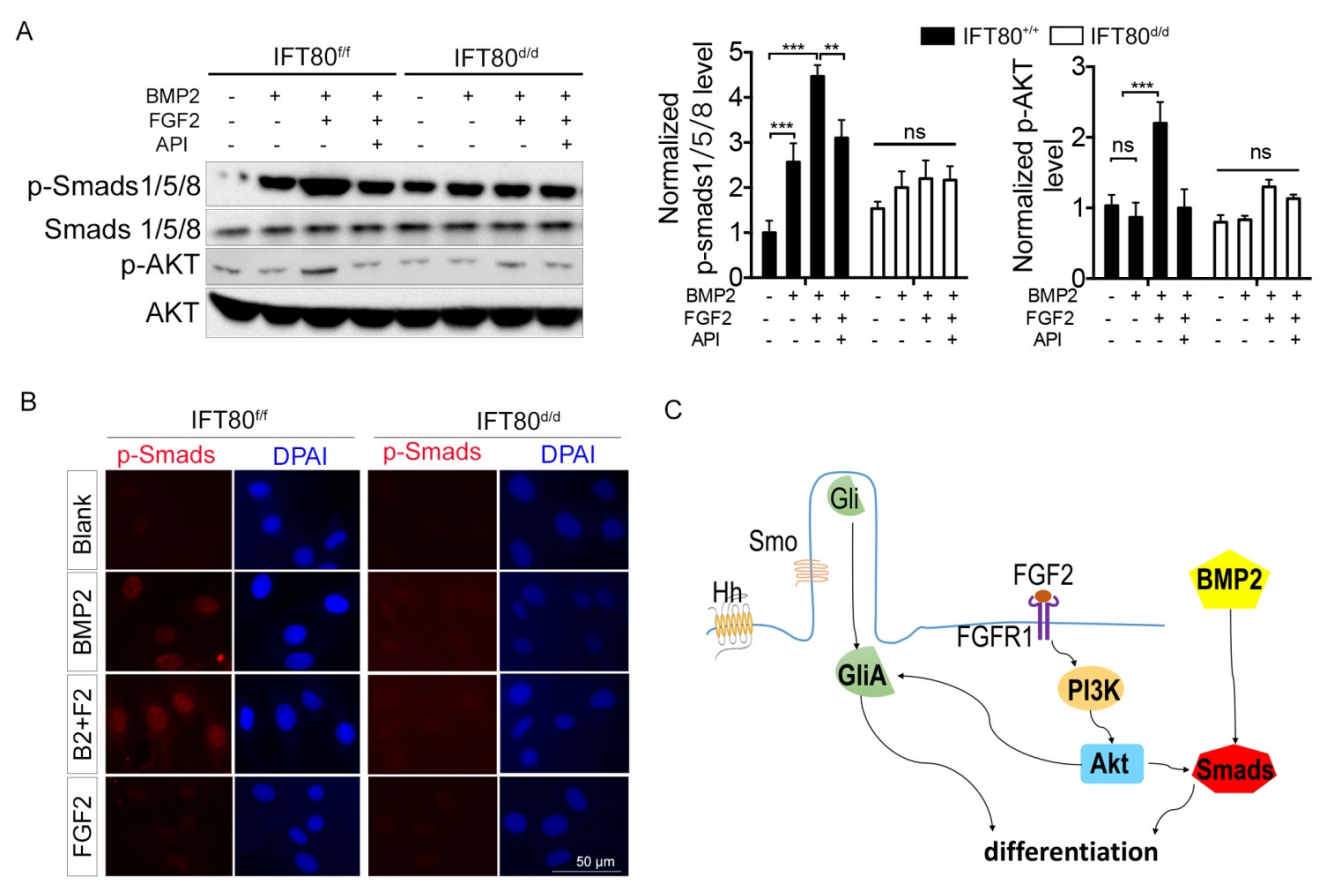
** Crosstalk between FGF2 and Hh/BMP2 signaling during DPSC differentiation is blocked in *IFT80*-deficient DPSCs.** (A) Western blot analysis of Smads1/5/8 and AKT phosphorylation in *IFT80^f/f^* and *IFT80^d/d^* DPSCs. Cells were treated with BMP2 (100 ng/mL), FGF2 (10 ng/mL), and API-2 (API, 1 μM) as indicated. Phosphorylation of Smads1/5/8 and AKT was normalized to Smads1/5/8 and AKT, respectively (n = 3). (B) Immunofluorescence analysis of p-Smads1/5/8 in *IFT80^f/f^* and *IFT80^d/d^* DPSCs (red). DAPI staining was used as counterstaining. Scale bars represent 50 μm. (C) Schematic representation of the proposed function of IFT80/cilia in FGF2 signaling, Hh signaling, and their crosstalk. Data are expressed as mean ± SEM; ns, not statistically significant; **p < 0.01; ***p < 0.0001.

To further uncover the crosstalk among FGF2, Hh and BMP2, we examined Smads1/5/8, the critical downstream mediator of BMP2 signaling, using both Western blot and immunostaining method. We found that FGF2 aided phosphorylation of Smads1/5/8 (Fig. 7A) and nuclear translocation of p-Smads1/5/8 (Fig. 7B). PI3K-AKT signaling is essential for the activity of BMP2 signaling 29, and our results showed that FGF2 could activate PI3K-AKT cascade (Fig. 7A), demonstrating FGF2 promoted BMP2 signaling through PI3K-AKT signaling. Impaired FGF2-PI3K-AKT transduction in *IFT80^d/d^* DPSCs disrupted synergistic effect with BMP2 signaling. Indeed, BMP2 activated phosphorylation of Smads1/5/8 and BMP2 induced p-Smads1/5/8 nuclear translocation also impaired in *IFT80^d/d^* DPSCs (Fig. 7A and 7B). Overexpression of BMP2 by transfection partially rescued the differentiation of *IFT80^d/d^* DPSCs (Fig. S4A and S4B). However, BMP2 did not change the cilia length and ciliated cell population (Fig. S4C). Smad1 linker phosphorylation is an essential downstream of FGF signaling to antagonist BMP2 signaling 30, 31, as we expected, impaired FGF signaling in *IFT80^d/d^* DPSCs caused impaired Smad linker phosphorylation compared with that in *IFT80^f/f^* DPSCs (Fig. S5).

## Discussion

DPSCs give rise to odontoblasts that produce dentin. Primary cilia have been observed in both DPSCs and odontoblasts more than a decade ago 11; however, the roles of primary cilia and ciliary proteins in DPSC differentiation and function were not well-addressed. Our study reveals for the first time that IFT80 is required for DPSC differentiation. Deletion of *IFT80* in DPSCs reduces FGFR1 expression and consequently impairs FGF2-FGFR1-PI3K-AKT signaling. Moreover, *IFT80* deletion impairs cilia formation, Hh signaling, as well as the coupling of FGF2 and Hh/BMP2 signaling, which eventually blocks DPSC differentiation.

The primary cilium is a signaling hub in a cell 17, 32, 33. Excepted for the well-known Hh signaling and Wnt signaling, emerging studies have suggested that IFT proteins and primary cilia mediate platelet-derived growth factor (PDGF) signaling 34, Notch 35, transforming growth factor beta (TGF-β), insulin-like growth factor (IGF) 36, 37, mammalian target of rapamycin (mTOR) 33, and epidermal growth factor (EGF) 32 signaling pathways. The role of primary cilia in FGF signaling was not well-defined. It has been reported that mutation of FGFR1, a receptor of FGF signaling, in zebrafish shortens cilia, implying signaling through FGFR1 is required for cilia formation 38. Since FGF signaling is also a critical signaling pathway for tooth development, we hypothesized that primary cilia/IFT proteins could regulate FGF signaling in DPSC differentiation. We found that loss of *IFT80* in DPSCs dramatically reduced FGFR1 expression (Fig. 3). FGFR1 or p-FGFR1 was not accumulated in cilia even with FGF2 stimulation (Fig. 4), suggesting cilia loss was not a direct cause for the reduced FGFR1 expression in *IFT80*-deficient DPSCs. One possible explanation is that the FGFR1 reduction results from the disruption of Hh-Gli signaling in *IFT80*-deficient DPSCs. This hypothesis was supported by the study from Qin et al. 39, showing inhibition of Gli with Gli-antagonist 61 (GANT61), a Gli specific inhibitor, inhibits FGFR1 expression. Another possibility is FGFR1 expression is regulated by ciliary proteins that are impaired in *IFT80*-deficient DPSCs. Centrin-2, a small calcium-binding protein, might be one of these cilia-associated proteins that regulated by IFT80. Centrin-2 is required for FGF8 and FGFR1 expression in Xenopus laevis because it is associated with their RNA polymerase II binding site 40. Since Centrin-2 localizes in the basal body of cilia 40, its function or expression might be affected by IFT80 and cilia loss. It is also possible that proteins involved in FGFR1 degradation are associated with primary cilia. The exact mechanism of how IFT80 regulates FGFR1 expression still needs further study.

The role of FGF2 in osteogenic differentiation is quite controversial 41 and seems stage-specific as suggested by recent studies 7, 42. Early and limited FGF2 treatment promotes differentiation through FGFR-MEK-ERK1/2 and BMP signaling pathway, whereas continuous FGF2 treatment inhibits mineralization 7. In our study, we confirmed that FGF2 has stimulatory effects on DPSC differentiation at an early stage beside its proliferation effect. More importantly, we found that FGF2 treatment significantly promotes cilia elongation and Hh signaling activation in DPSCs. It is well established that Hh signaling transduction requires functional cilia. Thus, pretreating DPSCs with FGF2 modulates cilia formation (Fig. 5), which aids Hh signaling activation (Fig. 6C). The exact mechanism of FGF2 in cilia formation is not entirely clear. Several recent studies demonstrated the regulatory role of actin cytoskeleton in ciliogenesis in different types of cells 43-46. Cytoskeleton remodeling shows both direct and indirect effects in ciliogenesis. For example, inhibiting branched actin network or actin destabilization directly facilitates ciliogenesis 45, 46 and cytoskeleton remodeling could also modulate transcriptional coactivator such as YAP/TAZ to regulate ciliogenesis 44. Our data demonstrated FGF2 causes cytoskeleton reorganization in DPSCs, and most likely the cytoskeleton remodeling promotes cilia formation in DPSCs.

We also found FGF2 activated PI3K-AKT (Fig. 6C) to promote Hh signaling, which is supported by the findings from Riobo et al. that Akt promotes Shh signaling by controlling PKA-mediated Gli inactivation 47. In addition to Hh signaling, we found FGF2 also promoted BMP2 signaling, an important differentiation signaling pathway. FGF2 signaling, as well as Hh signaling, stimulated BMP2 expression, and the combination of FGF2 and Hh signaling showed a synergistic effect. Similar to Hh activation, BMP2 signaling is greatly advanced by AKT activation 29. BMP2 itself could not activate AKT (Fig. 7A); therefore, FGF2-induced AKT activation serves as an enhancer for BMP2 expression, and then BMP2-induced Smads1/5/8 activation (Fig. 7A and 7B) and DPSC differentiation. Besides the activation effect, FGF2 signaling also promotes the phosphorylation of Smad1 linker to antagonist BMP2 signaling 30, 31. Although this negative feedback was blocked in IFT80^d/d^ DPSC, the overall effect still favors BMP2 signaling inhibition, eventually leading to the impaired DPSC differentiation.

Collectively, this study demonstrated that IFT80 is a critical regulator for DPSC differentiation. IFT80 maintains cilia and is required for FGFR1 expression. At the same time, FGF2/FGFR1-induced stress fiber rearrangement and AKT activation aid Hh signaling and BMP2 signaling activation to drive DPSC differentiation. In *IFT80*-deficient DPSC, FGF2/FGFR1 signaling, Hh/BMP2 signaling, and their coupling are impaired, which eventually leads to a differentiation defect. Thus, we revealed a novel role and mechanism of IFT80 in the regulation of DPSC differentiation and provided new insights for bone and tooth regenerative therapeutic design and therapy.

## Materials and Methods

### Mice

All experiments performed on mice were approved by the University at Buffalo Institutional Animal Care and Use Committee. The generation of *IFT80^f/f^* mice model (two LoxP sites flanking exon 6 of *IFT80*) was previously described 25.

*CiliaGFP* mice 48 were mated with *CMV-Cre* mice 49 to generate *CMV;CiliaGFP* mice, in which all the cilia are labeled with GFP. *CiliaGFP* mice were also mated with *IFT80^f/f^* mice to generate *CiliaGFP;IFT80^f/f^* mice.

### Regents

Recombinant mouse Shh N-terminus (1 μg/mL, R&D systems, Minneapolis, MN) or Purmorphomine (2 μM, Tocris Bioscience, 4551) were used to activate Hh signaling. FGF2 (10 ng/mL, R&D systems, Minneapolis, MN) was used to activate FGF signaling. BMP2 (100 ng/mL) was used to activate BMP2 signaling. PD173074 (1 μM, Tocris 3044) was used to inhibit FGFR. LY294002 (15 μM, Sigma, L9908) and API-2 (1 μM, Tocris 2151), were employed to inhibit PI3K and AKT respectively.

### Histology

Mouse mandibles were excised, fixed with 10% natural buffered formalin (VWR International, West Chester, PA, USA), and decalcified in 10% EDTA (ethylenediaminetetraacetic acid, Thermo Fisher Scientific) for two weeks at 4 °C. Paraffin-embedded samples were sectioned.

### DPSC isolation, culture, and differentiation

The incisors were isolated from the mandibles that dissected from 6-week old *IFT80^f/f^* mice. Whole dental pulp was gently collected from the interior of the incisor and exposed to enzymatic digestion with collagenase type I (3 mg/mL) and dispase (4 mg/mL) for one hour at 37°C with shaking. The digested tissues were homogenized by repetitive pipetting, and the released cells were centrifuged at 200
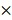
g for 10 minutes. The cells were cultured in alpha-modified Eagle's medium (α-MEM, Life Technologies) containing 10% fetal bovine serum (FBS, Life Technologies), 2 mM L-glutamine (Life Technologies), 100 U/mL penicillin and 100 μg/mL streptomycin (Life Technologies). DPSCs were allowed to adhere to the plastic dish for 24 hours, and then the medium was changed to remove floating debris. Culture medium was replaced every three days until the cells reach 80% confluence. Then the cells were detached by 0.25% Trypsin-EDTA (Life Technologies) and sub-cultivated at a ratio of 1:2 6, 50.

Odontogenic differentiation was induced with Odontogenic medium (OS medium) consisting of α-MEM (Gibico), 10% fetal bovine serum (FBS, Gibico), 10 mM β-glycerophosphate (Sigma), 50 μg/mL ascorbic acid (Sigma), 10^-8^ M dexamethasone (Sigma). ALP activity assay was performed 7 days after odontogenic induction. The cells were lysed with harvest buffer containing 10 mM Tris-Cl (pH 7.4), 0.2% NP40, and 2 mM PMSF. The lysates were homogenized and cleared by centrifuging. The supernatants were mixed with assay buffer (100 mM glycine and 1 mM MgCl_2_, pH 10.5) and p-Nitrophenyl Phosphate (PNPP) solution (50 mM in 0.1 M glycine buffer), and incubated at 37 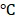 for 5-15 min. The reaction was stopped by the NaOH solution (0.1 N). The optical density was measured at 405 nm using AD 340 microplate reader (Beckman Coulter, Fullerton, CA). ALP activity was normalized to total cellular protein determined by BCA protein assay kit (Thermo Fisher Scientific) and expressed as units per minute per gram of total protein. To measure mineralization, Alizarin Red S staining was performed either 14 days or 21 days after odontogenic induction. The cells were washed with PBS, fixed with 2% formaldehyde, and then stained with 40 mM of Alizarin Red S solution (pH 4.2) at room temperature for 30 min. Cells were rinsed five times with dH_2_O to reduce nonspecific staining. Quantification was performed with a destaining solution containing 10% cetylpyridinium chloride and 10 mM sodium phosphate (pH 7.0) and measured at the wavelength of 562 nm. The experiment was done in triplicate.

Adipogenesis was induced with adipogenic medium containing α-MEM supplemented with 10% FBS, 10^-7^ M dexamethasone (Sigma), and 5
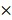
10^-6^ M insulin (Sigma). To examine adipogenesis, Oil Red O staining was performed. The cells were fixed with 10% formaldehyde for 10 min and stained with Oil Red O solution (Sigma). Hematoxylin was used as the counterstain.

Chondrogenesis was induced with chondrogenic medium containing DMEM supplemented with 10% FBS and ITS supplement (insulin-10 μg/mL, transferrin-5.5 μg/mL, and sodium selenite-5 ng/mL, Sigma-13146) for three weeks. To examine chondrogenesis, Alician Blue staining was performed. The cells were fixed with 4% glutaraldehyde for 15 min, stained with an acidic solution containing 1% Alician Blue (Sigma) for 30 min at room temperature and washed three times with hydrochloric acid 0.1 N.

DPSCs from *IFT80^f/f^* mice were infected with the adenovirus that overexpresses either Cre (Ad-CMV-Cre, #1405, Vector Biolabs) or GFP (Ad-GFP, #1060, Vector Biolabs). The Ad-CMV-Cre infection causes *IFT80* deletion in *IFT80^f/f^* DPSCs, which were then marked as *IFT80^d/d^*. Ad-GFP was used as an infection control and Ad-GFP treated *IFT80^f/f^* DPSCs were still marked as *IFT80^f/f^*. DPSCs from *CiliaGFP* mice were infected with Ad-CMV-Cre to turn on GFP express to mark cilia. DPSCs were infected with Ad-BMP2 to overexpress BMP2.

### Western blot

Cells were harvested and homogenized with RIPA buffer (50 mM Tris, 150 mM NaCl, 1% Triton X-100, 0.1% SDS, 1% sodium deoxycholate, and protease inhibitor cocktail). Protein was denatured in sodium dodecyl sulfate (SDS) buffer and separated with SDS-PAGE gels. Proteins were transferred to polyvinylidene difluoride (PVDF) membranes and then blocked with 5% skim milk (OXOID). Membranes were incubated with primary antibody overnight at 4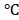, and then incubated with horseradish peroxidase (HRP) conjugated goat anti-rabbit IgG antibody (1:10000, Novex, Carlsbad, CA) at room temperature for 1 hour. Visualization was performed with WesternBright ECL HRP (Bio-Rad). β-actin (1:500, Santa Cruz) was used as the internal control.

The same procedure was used to determine the IFT80 (1:400, PAB15842, Abnova), FGFR1 (1:1000, ab10646, Abcam), p-FGFR1 (1:1000, ab59194, Abcam), Smad1/5/8 (1:300, sc-6031-R, Santa Cruz), p-Smad1/5/8 (1:300, sc-12353-R, Santa Cruz), AKT (1:300, sc-8312, Santa Cruz), p-AKT (1:300, sc-7985-r, Santa Cruz).

### qPCR

Total RNA was extracted from cultured DPSCs with Trizol reagent (Invitrogen, Carlsbad, CA) and then synthesized to cDNA with total RNA by RNA to cDNA EcoDry Premix kit (Clontech, Palo Alto, CA). qPCR was performed with ABI PRISM 7500 real-time PCR machine (Invitrogen, Carlsbad, CA) and SYBR Green PCR Master Mix (Invitrogen). Sequence and product length for each primer pair were listed in Supplementary Table S1. Gene expression was normalized to the housekeeping gene GAPDH and calculated according to the 2^-ddCT^ method 51. All reactions were run in triplicate.

### Immunocytochemistry and Immunofluorescence

Deparaffinized sections or fixed cells were permeabilized, blocked and incubated with acetylated α-tubulin (1:200, T6793, Sigma) overnight at 4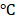. The slides were washed and stained with Alexa Fluor 568 conjugated anti-rabbit IgG (1:1000, Invitrogen) antibody for 1 hour at room temperature. DAPI (6-diamidino-2-phenylindole, Sigma) staining was used as the counterstain for nuclei.

The same staining procedure was used for acetylated α-tubulin (1:500, T6793, Sigma), FGFR1 (1:100, ab10646, Abcam), p-FGFR1 (1:100, ab59194, Abcam), Phospho-SMAD1 (Ser206) (1:100, PA517092, Invitrogen), and p-Smad1/5/8 (1:50, sc-12353-R, Santa Cruz) staining.

### Statistical analysis

All data are presented as mean ± SEM (n = 3 or more as indicated in figure legends). Comparisons between two groups were performed by Student's *t*-test and comparisons among grouped samples were analyzed by two-way ANOVA followed by Tukey's multiple comparisons. *P<0.05* was considered to be of statistical significance. The program GraphPad Prism (GraphPad Software, Inc., San Diego, USA) was used for these analyses.

## Supplementary Material

Supplementary figures and tables.
