# Supplementary material for: Ciliary IFT80 regulates dental pulp stem cells differentiation by FGF/FGFR1 and Hh/BMP2 signaling
Source: Int J Biol Sci. 2019 Aug 6;15(10):2087–99. doi: 10.7150/ijbs.27231 (PMC6775288; doi:10.7150/ijbs.27231)
Supplement: Supplementary file 1 — Supplementary figures and tables. [file ijbsv15p2087s1.pdf]

Supplemental information

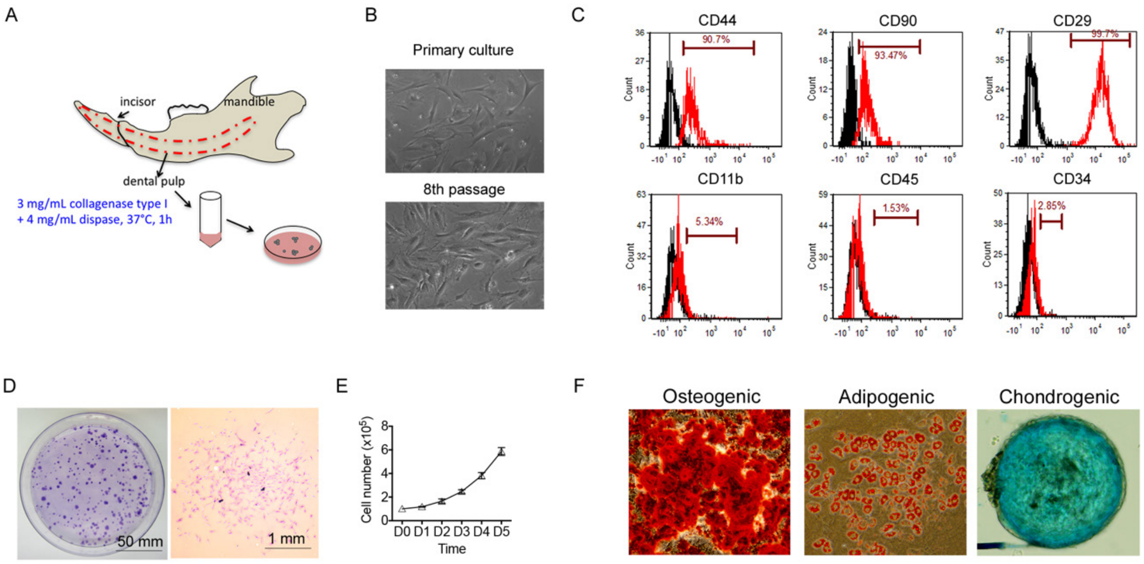

Figure S1. Characteristics of DPSCs. (A) Schematic representation showing DPSCs isolation from dental pulp of incisors. (B) Morphology of DPSCs in primary culture and 8th passage. Primary culture of DPSCs was heterogeneous with spindle-like, endothelial-like and epithelial-like shapes. The cell shapes become homogenous from passage 3 displaying only fibroblastic-like cells. (C) Immunophenotype of cultured DPSCs by the flow cytometric analysis. The red open histograms show that DPSCs are positive for the undifferentiated MSCs markers CD44, CD90, and CD29 but negative for monocyte/macrophage markers CD11b, CD45 and CD34. The black open histograms show isotype-matched control staining. (D) The colony formation capacity of DPSCs. The colonies with fibroblast-like DPSCs were visualized by Giemsa Stain. DPSCs displayed 123.9 ± 6.84 colonies per 1000 cells. (E) Growth curve of DPSCs at passage 6. The doubling time of DPSCs is 43.3 ± 3.4 h. (F) Differentiation potential of DPSCs. DPSCs were induced under odontogenic, adipogenic, or chondrogenic conditions. Calcium deposition in odontogenic differentiation was detected by Alizarin Red staining. Small lipid droplets in the cytoplasm of adipogenic differentiation were detected by Oil Red O staining. Aggrecan in chondrogenic differentiation was stained by Alician Blue.

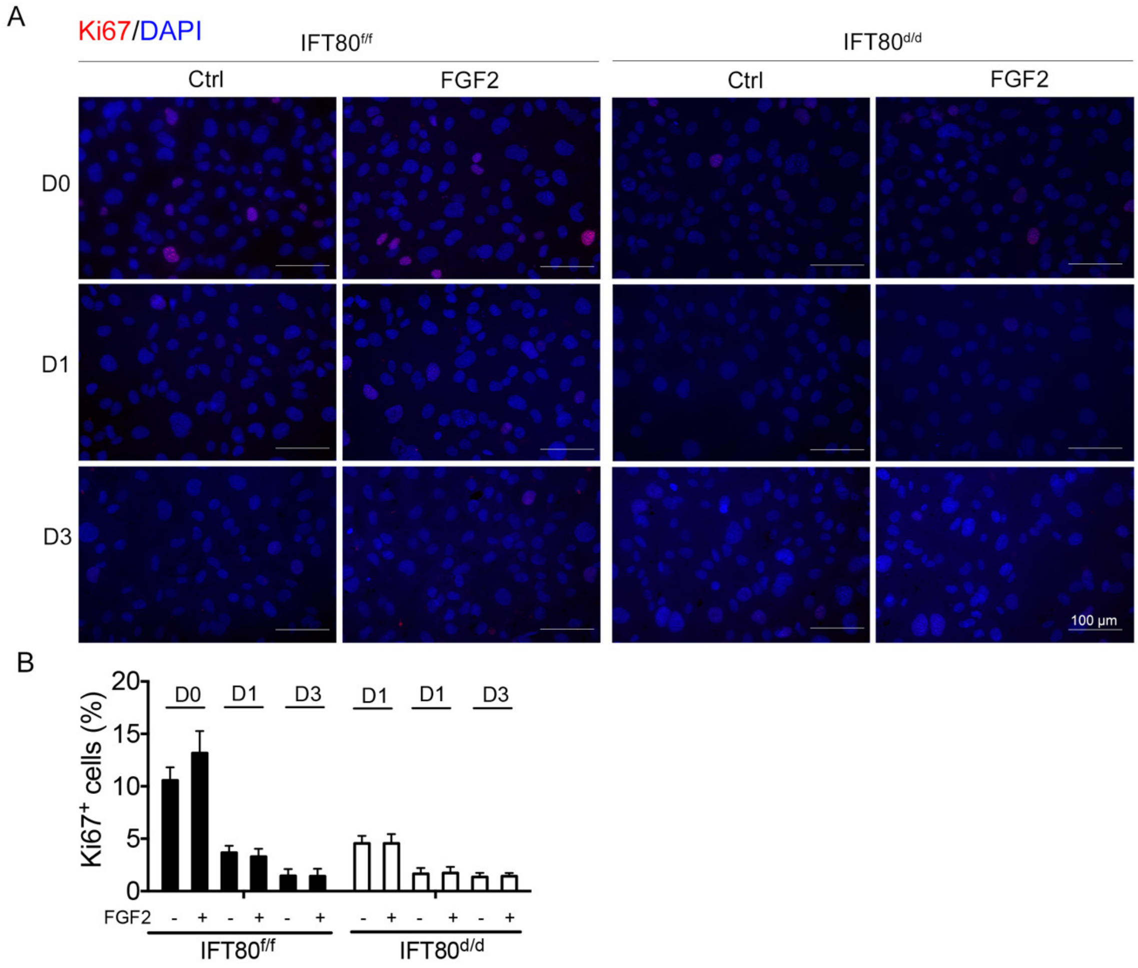

Figure S2. Proliferation rate is at minimum level during differentiation. (A) Ki67 (red) staining of *IFT80*$^{f/f}$ and *IFT80*$^{d/d}$ DPSCs during differentiation. *IFT80*$^{f/f}$ and *IFT80*$^{d/d}$ DPSCs were seeded at the density of 1000 cells per mm^2 (subconfluent) and induced with OS medium with or without FGF2 (10 ng/mL). Ki67 staining was performed at D1, D2, and D3. DAPI staining was used as a counterstaining to calculate the total cell numbers. Scale bars represent 100 µm. (B) Calculated the percentage of Ki67 positive cells at indicated conditions (n=3 with at least 500 cells analyzed).

Data are expressed as mean ± SEM.

A

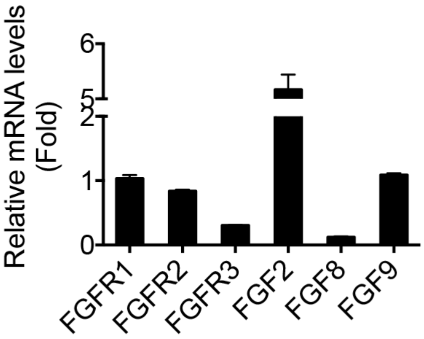

Figure S3. *FGFRs* and *FGFs* expression in DPSCs. (A) qPCR analysis of *FGFR1*, *FGFR2*, *FGFR3*, *FGF2*, *FGF8* and *FGF9* expression in DPSCs (n = 3, triplicates per group). Data are expressed as mean ± SEM.

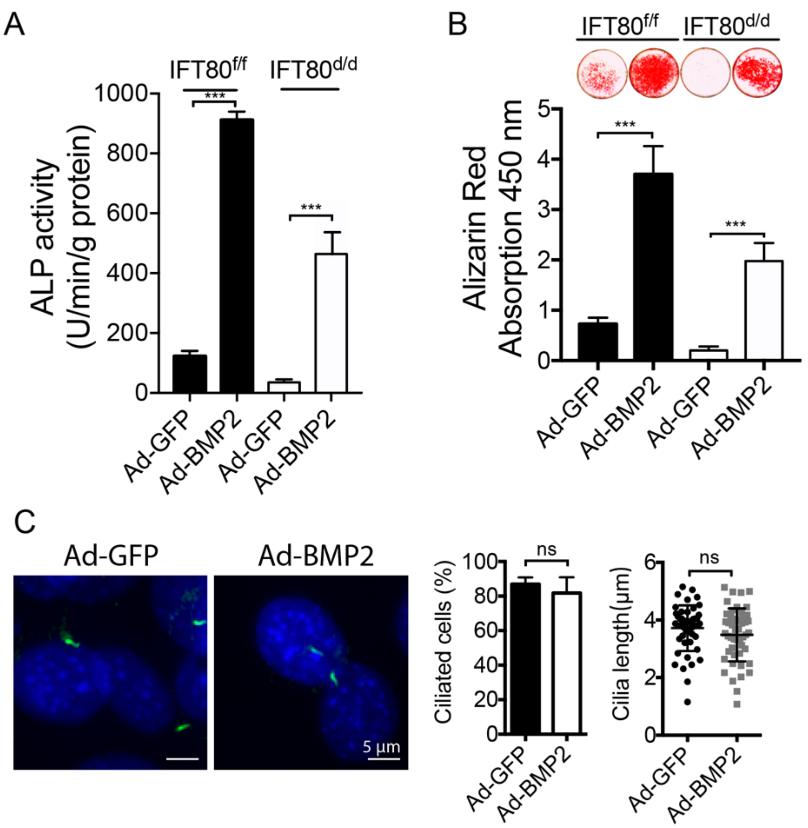

Figure S4. Overexpression of BMP2 partially rescues the differentiation of *IFT80$^{d/d}$* DPSCs.

(A) ALP activity of *IFT80$^{f/f}$* and *IFT80$^{d/d}$* DPSCs at day 7 of OS induction transfected with Ad-GFP (control) or Ad-BMP2 (n = 3, triplicates per group). (B) Alizarin Red staining of *IFT80$^{f/f}$* and *IFT80$^{d/d}$* DPSCs at day 14 of OS induction transfected with Ad-GFP (control) or Ad-BMP2 (n = 3, triplicates per group). (C) Immunofluorescence analysis of primary cilia in cultured DPSCs. Primary cilia were stained with acetylated α-tubulin (green) antibody. DAPI staining was used for counterstaining. Scale bars represent 5 μm. Cilia length (n = 20 cells) and cilia percentage (n = 3 with at least 200 cells analyzed) were calculated.

Data are expressed as mean ± SEM; ns, not statistically significant; ***p < 0.0001.

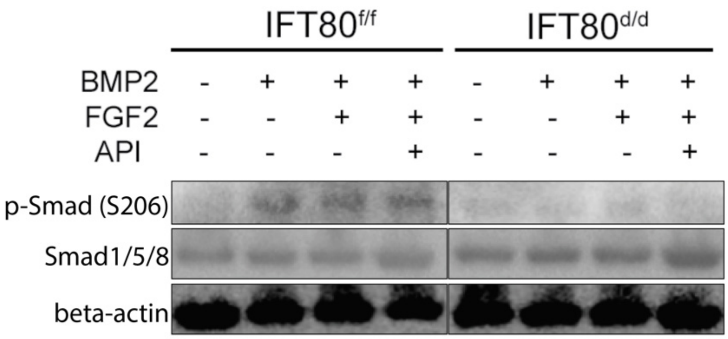

Figure S5. Smad1 linker phosphorylation is damaged in *IFT80$^{d/d}$* DPSCs*.* Western blot analysis of smad1 linker phosphorylation (S206) in *IFT80$^{f/f}$* and *IFT80$^{d/d}$* DPSCs. Cells were treated with BMP2 (100 ng/mL), FGF2 (10 ng/mL), and API-2 (API, 1 μM) as indicated. Smads1/5/8 and beta-actin were served as internal controls.

Supplementary table 1. List of primers used in this study.

Gene	Forward primer sequence	Reverse primer sequence	Length
IFT80	AAGGAACCAAAGCATCAAGAATTAG	AGATGTCATCAGGCAGCTTGAC	148 bp
FGFR1	AGACTCCACTTCCACAGGGA	CCAACCTCTAACCGCAGAAC	150 bp
FGFR2	CGCTGTAAACCTTGCAGACA	GGAGAATGAATACGGGTCCA	149 bp
FGFR3	GCATCCTCACTGTGACATCAAC	CCTGGCGAGTACTGCTCAAA	70 bp
FGF2	GCTGCTGGCTTCTAAGTGTGT	TCTGTCCAGGTCCCGTTTTG	161 bp
FGF8	GTGGAGACCGATACTTTTGG	GCCCAAGTCCTCTGGCTGCC	371 bp
FGF9	ATGGCTCCCTTAGGTGAAGTT	TCCGCCTGAGAATCCCCTTT	190 bp
Gli1	GGTCTCGGGGTCTCAAACTG	CCATTCTCTGGTGGGGTTCC	184 bp
Ptch1	GACCGGCCTTGCCTCAACCC	CAGGGCGTGAGCGCTGACAA	204 bp
DMP1	GCTTCAGGCTCAGTTTTGCT	TGTAACCCTCCAGCTCCAGG	258 bp
DSPP	GGCCAATCTCATGGGGGAAA	GAGCTTTTGGTTGTCCTGCG	177 bp
GAPDH	TGTGTCCGTCGTGGATCTGA	TTGCTGTTGAAGTCGCAGGAG	150 bp
